# Functional Characterization of Variants of Unknown Significance of Fibroblast Growth Factor Receptors 1-4 and Comparison With AI Model–Based Prediction

**DOI:** 10.1200/PO-24-00847

**Published:** 2025-06-17

**Authors:** Martin Ziegler, Nadira Khoury, Louisa Maxine Hommerich, Heike Adler, Sonja Loges

**Affiliations:** ^1^ DKFZ-Hector Cancer Institute at the University Medical Center Mannheim, Mannheim, Germany; ^2^ Division of Personalized Medical Oncology (A420), German Cancer Research Center (DKFZ); German Center for Lung Research (DZL), Heidelberg, Germany; ^3^ Department of Personalized Oncology, University Hospital Mannheim, Medical Faculty Mannheim, University of Heidelberg, Mannheim, Germany

## Abstract

**PURPOSE:**

Fibroblast growth factor receptors (FGFRs; FGFR1, FGFR2, FGFR3, FGFR4) are frequently mutated oncogenes in solid cancers. The oncogenic potential of FGFR rearrangements and few hotspot point mutations is well established, but the majority of variants resulting from point mutations especially outside of the tyrosine kinase domain are currently considered variants of unknown significance (VUS).

**MATERIALS AND METHODS:**

Recurrent nonkinase domain FGFR VUS variants were collected from the Catalog of Somatic Mutations in Cancer and their oncogenic potential was assessed in vitro by different functional assays. We compiled published clinical and preclinical data on FGFR variants and compared the data with results from our functional assays and pathogenicity predictions of state-of-the-art artificial intelligence (AI) models.

**RESULTS:**

We identified 12 novel FGFR extracellular small variants with potential driver function. Comparison of clinical and preclinical data on FGFR variants with pathogenicity predictions of state-of-the-art AI models showed limited usefulness of the AI predictions. Sensitivity profiles of activating FGFR variants to targeted inhibitors were recorded and showed good targetability of FGFR nonkinase domain variants.

**CONCLUSION:**

The collected results extend the spectrum of suitable FGFR variants for potential treatment with FGFR inhibitors in the context of clinical trials and beyond. Current AI models for variant pathogenicity prediction require further refinement for use in oncogenic decision making.

## INTRODUCTION

**FIG 1.**
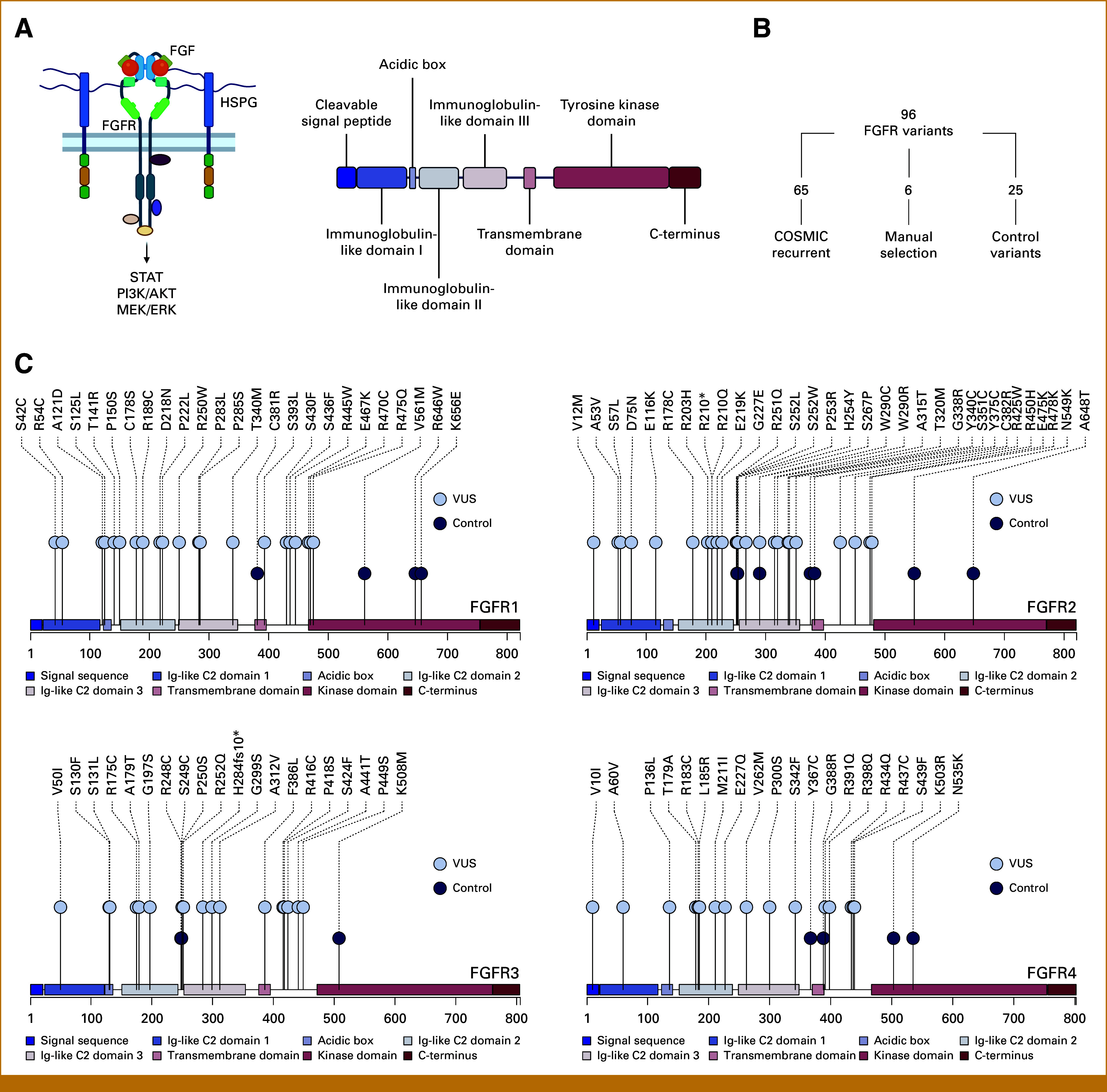
(A) Overview of FGFR signaling (left) and schematic of the major functional regions of FGFRs (right). (B) Composition of the investigational set of FGFR VUS. (C) Lolliplots showing the distribution of FGFR variants studied in this work. COSMIC, Catalog of Somatic Mutations in Cancer; FGF, fibroblast growth factor; FGFR, fibroblast growth factor receptor; HSPG, heparan sulfate proteoglycan; VUS, variant of unknown significance.

Fibroblast growth factor receptors (FGFRs) are a family of transmembrane receptor tyrosine kinases involved in cellular signaling during development and in adult tissue homeostasis.^1^ Signaling is initiated by binding of fibroblast growth factors and cofactors to the extracellular domains of FGFRs and transmitted via receptor dimerization, trans-auto-phosphorylation of FGFR kinase domains, and activation of intracellular downstream signaling targets (Fig 1A). Ultimately, important biological functions such as proliferation, differentiation, angiogenesis, and wound healing are modulated.^2,3^

**CONTEXT**

**Key Objective**
Which additional fibroblast growth factor receptor (FGFR) mutations located outside of the tyrosine kinase domain exert activating effects and are potentially targetable by FGFR tyrosine kinase inhibitors (TKIs)?
**Knowledge Generated**
Twelve novel nonkinase domain FGFR mutations that have not yet been investigated in clinical trials demonstrated at least moderately activating potential in vitro. The identified FGFR mutations are generally well targetable with available approved or investigational FGFR TKIs.
**Relevance**
The newly described activating FGFR mutations could be considered for clinical evaluation in clinical trials, which may potentially allow to expand the set of targetable FGFR aberrations in future.

Approximately 5%-10% of solid tumors carry mutations in one of the four FGFRs, and aberrant FGFR signaling can be involved in oncogenesis and cancer progression.^4^ Oncogenic activation of FGFRs occurs through small variants, genomic rearrangements, and amplification. Following successful clinical trials, several FGFR targeting drugs have been approved by the Food and Drug Administration for clinical use: erdafitinib in advanced refractory urothelial carcinoma for tumors harboring FGFR2 or FGFR3 fusions or selected FGFR3 point mutations,^5^ futibatinib in advanced cholangiocarcinoma with FGFR2 rearrangements,^6^ and pemigatinib in advanced cholangiocarcinoma with FGFR2 rearrangements^7^ and in relapsed or refractory myeloid-lymphoid neoplasms with FGFR1 rearrangements.^8^ The FGFR tyrosine kinase inhibitor (TKI) infigratinib had also received accelerated approval for advanced cholangiocarcinoma bearing FGFR2 rearrangements,^9,10^ but the conditional approval has been withdrawn after cessation of clinical trials by the sponsor. Next to FGFR-specific inhibitors, reports from case studies or pilot studies also demonstrate potential clinical efficacy of several multikinase inhibitors cotargeting FGFRs such as lenvatinib, nintedanib, pazopanib, and ponatinib in FGFR-altered tumors.^11-17^

Recent entity-agnostic basket trials for erdafitinib and pemigatinib have considerably expanded the spectrum of tumor entities with potential clinical benefits from FGFR-targeted therapy in molecularly stratified cohorts.^18-20^ At the present time, molecular inclusion criteria encompass FGFR2 or FGFR3 rearrangements and few well-characterized hotspot mutations in the extracellular domains of FGFR2 and FGFR3. However, large-scale sequencing efforts have demonstrated that the spectrum of clinically observed FGFR variants is much broader. Data from The Cancer Genome Atlas show a plethora of low-frequency point mutations scattered across the coding regions of FGFR genes (Data Supplement, Fig S1). In most entities, these scattered mutations considerably outnumber known hotspot mutations. The functional consequences of the majority of the resulting small variants remain unknown due to lack of experimental and clinical data. Existing studies indicate that activating hotspot mutations inside and outside of the kinase domains of FGFRs are generally targetable by FGFR-TKIs, but resistance mutations are typically restricted to the kinase domains.^21,22^ Nonkinase FGFR mutations outside of hotspots thus form a potentially well-targetable but insufficiently characterized group of FGFR alterations.

To address the need for characterization of scattered FGFR variants and potentially expand the spectrum of targetable FGFR alterations, we collected nonkinase domain FGFR variants from the Catalog of Somatic Mutations in Cancer (COSMIC).^23^ We assessed their oncogenic potential and sensitivity to FGFR-TKIs in vitro and compared the results with predictions generated by state-of-the art artificial intelligence (AI) prediction tools. We report the discovery of 12 novel activating nonkinase domain FGFR variants with mostly moderately activating effects and concomitant sensitivity of cell lines expressing these variants to FGFR targeting inhibitors. Furthermore, we classified 59 FGFR point mutations outside of the tyrosine kinase domain as nonactivating.

## MATERIALS AND METHODS

### Cohort Collection

FGFR variants were collected from COSMIC (accessed on July 11, 2022). Recurrent small variants (single amino acid exchanges or indels) on protein level occurring N-terminally of the FGFR kinase domains with at least three counts in COSMIC formed the initial candidate set. We filtered out variants that had been compellingly characterized in previous studies, only keeping variants with unknown functional effects, and variants with conflicting or unclear data from existing studies. We complemented the investigational set with six manually selected variants (based on functional and/or structural properties) and 25 mutations with known activating, inactivating, or resistance-conferring function. The final set contained 96 variants. The R-package trackViewer was used for visualization.^24^

### Reagents and Cell Culture Procedures

Reagents used in this study are listed in the Data Supplement (Table S1). NIH/3T3 were routinely cultured in RPMI 1640 + GlutaMAX supplemented with 10% bovine serum (BS). Phoenix Eco were routinely cultured in DMEM + GlutaMAX supplemented with 10% fetal calf serum. All cell lines were authenticated and tested for mycoplasma infection. The Data Supplement (Table S2) lists all cell lines used in this study.

### Cloning and Cell Line Engineering

Plasmids and primers used in this study are listed in the Data Supplement (Tables S3 and S4). cDNAs for expression of wild-type FGFR1, FGFR2, FGFR3, and FGFR4 were cloned from the source plasmids into EcoRI-digested pMIGR1 using Gibson Assembly. Deviations from reference transcripts (FGFR1: NM_023110.3, FGFR2: NM_000141.5, FGFR3: NM_000142.5, FGFR4: NM_213647.3) were corrected by site-directed mutagenesis using Q5 Site-Directed Mutagenesis Kit. Mutations coding for FGFR variants were subsequently individually introduced by site-directed mutagenesis and verified by sequencing.

Phoenix Eco were transfected with plasmids carrying FGFR variants using Lipofectamine 2000. Supernatant containing ecotropic retrovirus particles was harvested 2-3 days after transfection, sterile-filtered, and used for infection of NIH/3T3 assisted by 4 μg/mL Polybrene. NIH/3T3 carrying FGFR expression constructs were then sorted to purity based on translationally coupled enhanced green fluorescent protein expression.

### Low-Serum Proliferation Assay

NIH/3T3 expressing FGFR variants were seeded into 96-well plates at a density of 5,000 cells per well in RPMI 1640 + GlutaMax supplemented with 1% penicillin/streptomycin and serum concentrations ranging from 0% to 5% BS. After incubation for 7 days, the viability of cells was measured with ATPlite 1step Luminescence Assay System. In validation experiments, the incubation time for selected FGFR variants with strong level of activation was shortened to 4 days to prevent premature loss of viability due to cellular overgrowth. Data were background-corrected, normalized to the signal obtained from wells with 5% BS, and the R package drc^25^ was used to analyze data with a two-parameter log-logistic dose-response model.

### TKI Sensitivity Profiling

NIH/3T3 expressing FGFR variants were seeded into 384-well plates at a density of 1,200 cells per well in RPMI 1640 + GlutaMax supplemented with 1% penicillin/streptomycin and 1.5% BS. Ten millimolar inhibitor stock solutions in dimethylsulfoxate (DMSO) were serially diluted and added to achieve final concentrations ranging from 0.085 nM to 5,000 nM. After incubation for 5 days, the viability of cells was measured with the ATPlite 1step Luminescence Assay System. Data were background-corrected, normalized to the signal obtained from wells with no inhibitor, and analyzed with the R package drc.^25^ For modeling purposes, blank DMSO controls were assigned to virtual concentrations of 0.001 nM, and four-parameter log-logistic dose-response models were calculated to estimate IC50 values. If dose-response relationships with biphasic patterns were observed, data points from the secondary plateau were omitted and we report the lower IC50 value. In cases with high inhibition already at the lowest experimental concentration used, a log-logistic dose-response model with fixed upper bound (100%) was calculated to enforce model fitting. To account for the reduced accuracy of drug-response modeling when IC50s are estimated close to border concentrations, IC50 values below 0.1 nM were reported as <0.1 nM and IC50 values exceeding 1,000 nM were reported as >1,000 nM. Cases in which IC50 estimation was not possible due to insufficient inhibition or in which models did not converge are reported as no inhibition/no model fit.

### Soft Agar Assay

A base layer of RPMI 1640 + GlutaMax supplemented with 1% penicillin/streptomycin and 10% BS containing 0.7% agarose was poured into six-well plates and solidified. NIH/3T3 expressing FGFR variants were seeded at a density of 2,500 cells per well into a semi-solid top layer containing 0.4% agarose. The top agar was covered with medium. For experiments with inhibitor, drugs were added to the top agar layer and the medium at a final concentration of 50 nM. After incubation for 14 days, colonies were stained with 1 mg/mL nitroblue tetrazolium chloride solution overnight, imaged, and the area covered by colonies was measured with the ImageJ Plugin ColonyArea.^26^ Area measurements from technical replicates were averaged and normalized to area measurements from control wells of NIH/3T3 expressing the corresponding FGFR wild-type proteins from the same plate.

### Analysis of FGFR Variants With Computational Predictive Models

AlphaMissense predictions were downloaded from Google cloud servers.^27^ CancerVar predictions were retrieved from the publicly available Web implementation at CancerVar.^29,59^ PrimateAI-3D predictions^28^ were provided by Illumina or accessed from the Illumina primAD browser Web implementation.^31^ The recommendation for a threshold of 0.8 was obtained from the primAD documentation.^32^ The predictions of all models were mapped to FGFR proteins produced from reference transcripts (FGFR1: NM_023110.3, FGFR2: NM_000141.5, FGFR3: NM_000142.5, FGFR4: NM_213647.3) and the scores for FGFR variants of unknown significance (VUS) collected. Mutations for which a model did not provide a prediction were not included in calculations of performance parameters. The clinical benefit data were collected from published articles as indicated in Table 1. Functional annotations based on preclinical data^30^ were collected from functional annotation of somatic mutations in cancer (FASMIC; accessed on August 26, 2024).^54^ CIs for sensitivity and specificity were estimated using the R package Hmisc 5.1.3 assuming a binomial distribution.^53^ Receiver operating characteristic (ROC) curves were created with the R package ROCplot.^55^

**TABLE 1.** FGFR Mutations in Reports of Clinical Cases With Benefit to FGFR TKI Treatment

FGFR	Mutation^a^	Entities	Best Clinical Response and Inhibitor^b^	Comments	Literature
FGFR1					
FGFR1	D60Y	Squamous cell lung cancer	Stable disease (AZD4547)	Complex mutation with FGFR1 H810Y^c^	^33^
FGFR1	S125L	Angiosarcoma	Stable disease (infigratinib)		^34^
FGFR1	A354V	Esophageal carcinoma	Partial response (pemigatinib)		^35^
FGFR1	I387V	Breast cancer	Stable disease (pemigatinib)	Complex mutation with FGFR3 R439C^c^	^20^
FGFR1	M456I	Not available	Stable disease (pemigatinib)		^20^
FGFR1	M532I	Anaplastic oligodendroglioma	Partial response (futibatinib)	Complex mutation with FGFR1 K656E^c^	^36^
FGFR1	M532T	Urothelial carcinoma	Partial response (pemigatinib)		^37^
FGFR1	M532T	Urothelial carcinoma	Partial response (futibatinib)		^38^
FGFR1	N546D	Glioblastoma	Partial response (futibatinib)		^36^
FGFR1	N546K	Head and neck cancer	Partial response (pemigatinib)		^39^
FGFR1	N546K	Low-grade glioneuronal tumor	Stable disease (erdafitinib)		^18^
FGFR1	V561M	Spinal cord high-grade glioma	Stable disease (zoligratinib)	Complex mutation with FGFR1 K656E^c^	^40^
FGFR1	V561M	Pilomyxoid astrocytoma	Stable disease (zoligratinib)	Complex mutation with FGFR1 K656E^c^	^40^
FGFR1	K656E	Central nervous system	Partial response (pemigatinib)		^20^
FGFR1	K656E	Low-grade glioma	Complete response (erdafitinib)		^19^
FGFR1	K656E	Low-grade glioma	Partial response (infigratinib)		^41^
FGFR1	K656E	Low-grade glioma	Stable disease (infigratinib)		^41^
FGFR1	K656E	Anaplastic oligodendroglioma	Partial response (futibatinib)	Complex mutation with FGFR1 M532I^c^	^36^
FGFR1	K656E	Spinal cord high-grade glioma	Stable disease (zoligratinib)	Complex mutation with FGFR1 V561M^c^	^40^
FGFR1	K656E	Pilomyxoid astrocytoma	Stable disease (zoligratinib)	Complex mutation with FGFR1 V561M^c^	^40^
FGFR1	E765G	Pilomyxoid astrocytoma	Partial response (zoligratinib)	Complex mutation with FGFR1-TACC3 fusion^c^	^40^
FGFR1	H810Y	Squamous cell lung cancer	Stable disease (AZD4547)	Complex mutation with FGFR1 D60Y^c^	^33^
FGFR2					
FGFR2	T98M	Cholangiocarcinoma	Stable disease (pemigatinib)		^20^
FGFR2	H167_N173del	Intrahepatic cholangiocarcinoma	Partial response (zoligratinib)	Two cases with similar outcome reported	^42^
FGFR2	H167_N173del	Intrahepatic cholangiocarcinoma	Partial response (futibatinib)	Complex mutation with FGFR1 L617F^c^	^42^
FGFR2	S252W	Salivary gland cancer	Stable disease (pemigatinib)		^39^
FGFR2	S252W	Gallbladder cancer	Stable disease (pazopanib)		^43^
FGFR2	S252W	Gallbladder cancer	Stable disease (erdafitinib)		^43^
FGFR2	S252W	Gynecologic	Stable disease (pemigatinib)	Two cases with similar outcome reported	^20^
FGFR2	S252W	Head and neck cancer	Partial response (erdafitinib)		^18^
FGFR2	S252W	Not available	Stable disease (AZD4547)	Three cases with similar outcome reported	^44^
FGFR2	F276C	Intrahepatic cholangiocarcinoma	Partial response (infigratinib)		^45^
FGFR2	F276C	Cholangiocarcinoma	Partial response (pemigatinib)		^35^
FGFR2	I288_E295delinsT	Intrahepatic cholangiocarcinoma	Partial response (zoligratinib)		^42^
FGFR2	I291_Y308del	Cholangiocarcinoma	Partial response (pemigatinib)		^20^
FGFR2	W290C	Cholangiocarcinoma	Stable disease (pemigatinib)		^20^
FGFR2	W290C	Intrahepatic cholangiocarcinoma	Partial response (futibatinib)		^38^
FGFR2	Y375C	Cholangiocarcinoma	Stable disease (pemigatinib)		^20^
FGFR2	Y375C	Endometrial cancer	Partial response (erdafitinib)		^19^
FGFR2	Y375C	Salivary gland cancer	Partial response (erdafitinib)		^19^
FGFR2	Y375C	Salivary gland cancer	Complete response (erdafitinib)		^19^
FGFR2	Y375C	Nonsquamous non–small cell lung cancer	Partial response (erdafitinib)		^19^
FGFR2	Y375C	Primary unknown	Partial response (futibatinib)		^38^
FGFR2	Y375C	Intrahepatic cholangiocarcinoma	Partial response (futibatinib)		^46^
FGFR2	Y375C	Intrahepatic cholangiocarcinoma	Partial response (AZD4547)		^44^
FGFR2	C382R	Head and neck carcinoma	Stable disease (pemigatinib)		^39^
FGFR2	C382R	Cholangiocarcinoma	Partial response (pemigatinib)		^39^
FGFR2	C382R	Gynecologic	Stable disease (pemigatinib)		^20^
FGFR2	C382R	Cholangiocarcinoma	Partial response (pemigatinib)		^20^
FGFR2	C382R	Endometrial cancer	Partial response (erdafitinib)		^19^
FGFR2	C382R	Breast cancer	Partial response (erdafitinib)		^19^
FGFR2	C382R	Cholangiocarcinoma	Partial response (erdafitinib)		^19^
FGFR2	C382R	Squamous-cell head and neck cancers	Partial response (erdafitinib)		^19^
FGFR2	C382R	Intrahepatic cholangiocarcinoma	Partial response (futibatinib)		^38^
FGFR2	C382R	Cholangiocarcinoma	Complete response (pemigatinib)		^47^
FGFR2	C382R	Endometrial cancer	Stable disease (erdafitinib)		^18^
FGFR2	V395D	Ameloblastoma	Partial response (erdafitinib)		^48^
FGFR2	N549D	Metastatic intrahepatic cholangiocarcinoma	Partial response (futibatinib)	Complex mutation with FGFR2-CORO2B fusion	^49^
FGFR2	N549S	Cholangiocarcinoma	Stable disease (infigratinib)		^34^
FGFR2	L617F	Intrahepatic cholangiocarcinoma	Partial response (futibatinib)	Complex mutation with FGFR1 H167_N173del^c^	^42^
FGFR2	E768fs	Not available	Stable disease (pemigatinib)		^20^
FGFR2	Q774*	Breast cancer	Stable disease (pemigatinib)		^20^
FGFR3					
FGFR3	R248C	Breast cancer	Partial response (erdafitinib)		^19^
FGFR3	R248C	Esophageal cancer	Partial response (erdafitinib)		^19^
FGFR3	R248C	Urothelial carcinoma	Partial response (pemigatinib)	Five cases with similar outcome reported	^50^
FGFR3	R248C	Urothelial carcinoma	Stable disease (pemigatinib)	Three cases with similar outcome reported	^50^
FGFR3	S249C	Urothelial carcinoma	Stable disease (pemigatinib)		^39^
FGFR3	S249C	Urothelial carcinoma	Partial response (pemigatinib)		^39^
FGFR3	S249C	Urothelial carcinoma	Partial response (pemigatinib)	Combination therapy with pembrolizumab	^37^
FGFR3	S249C	Not available	Stable disease (pemigatinib)	Two cases with similar outcome reported	^20^
FGFR3	S249C	Urothelial carcinoma	Stable disease (pemigatinib)	Four cases with similar outcome reported	^20^
FGFR3	S249C	Ovarian cancer	Partial response (erdafitinib)		^19^
FGFR3	S249C	Squamous non–small cell lung cancer	Partial response (erdafitinib)		^19^
FGFR3	S249C	Squamous cell head and neck cancer	Partial response (erdafitinib)	Two cases with similar outcome reported	^19^
FGFR3	S249C	Squamous cell lung cancer	Stable disease (AZD4547)	Parallel FGFR1 amplification	^33^
FGFR3	S249C	Urothelial carcinoma	Complete response (pemigatinib)	Two cases with similar outcome reported	^50^
FGFR3	S249C	Urothelial carcinoma	Partial response (pemigatinib)	24 cases with similar outcome reported	^50^
FGFR3	S249C	Urothelial carcinoma	Stable disease (pemigatinib)	42 cases with similar outcome reported	^50^
FGFR3	S249C	Urothelial carcinoma	Partial response (futibatinib)		^38^
FGFR3	S249C	Ovarian cancer	Partial response (erdafitinib)		^18^
FGFR3	S249C	Urothelial carcinoma	Complete response (erdafitinib)	Combination therapy with pembrolizumab	^51^
FGFR3	S249C	Not available	Stable disease (AZD4547)		^44^
FGFR3	S249C	Transitional cell carcinoma of renal pelvis	Stable disease (AZD4547)		^44^
FGFR3	S249C	Squamous cell lung cancer	Partial response (AZD4547)		^52^
FGFR3	S249C	Urothelial carcinoma	Partial response (infigratinib)	Four cases with similar outcome reported	^34^
FGFR3	S249C	Urothelial carcinoma	Stable disease (infigratinib)		^34^
FGFR3	S249C	Lung squamous cell carcinoma	Stable disease (infigratinib)		^34^
FGFR3	G370C	Not available	Stable disease (pemigatinib)		^20^
FGFR3	G370C	Urothelial carcinoma	Stable disease (pemigatinib)	Two cases with similar outcome reported	^20^
FGFR3	G370C	Urothelial carcinoma	Partial response (pemigatinib)	Two cases with similar outcome reported	^50^
FGFR3	G370C	Urothelial carcinoma	Stable disease (pemigatinib)		^50^
FGFR3	S371C	Not available	Stable disease (pemigatinib)		^20^
FGFR3	S371C	Urothelial carcinoma	Stable disease (pemigatinib)	Two cases with similar outcome reported	^50^
FGFR3	Y373C	Urothelial carcinoma	Partial response (pemigatinib)	Combination therapy with pembrolizumab	^37^
FGFR3	Y373C	Urothelial carcinoma	Partial response (pemigatinib)		^20^
FGFR3	Y373C	Urothelial carcinoma	Stable disease (pemigatinib)		^20^
FGFR3	Y373C	Urothelial carcinoma	Complete response (pemigatinib)		^50^
FGFR3	Y373C	Urothelial carcinoma	Partial response (pemigatinib)	Two cases with similar outcome reported	^50^
FGFR3	Y373C	Urothelial carcinoma	Stable disease (pemigatinib)	15 cases with similar outcome reported	^50^
FGFR3	F384L	Lung adenocarcinoma	Stable disease (infigratinib)	Three cases with similar outcome reported	^34^
FGFR3	A391E	Transitional cell carcinoma of urinary bladder	Partial response (AZD4547)		^44^
FGFR3	R439C	Breast cancer	Stable disease (pemigatinib)	Complex mutation with FGFR1 I387V^c^	^20^
FGFR3	K650E	Glioma	Stable disease (infigratinib)		^41^
FGFR3	K650E	Urothelial cancer	Stable disease (infigratinib)	Two cases with similar outcome reported	^34^

Abbreviations: FGFR, fibroblast growth factor receptor; TKI, tyrosine kinase inhibitor.

^a^ Mutations were mapped to the canonical isoforms (see Methods section).

^b^ Clinical benefit as reported in the source publication; if not available, best percentage change from baseline was used for inference: partial response was assumed for cases with at least 30% tumor reduction and stable disease for cases with not more than 30% tumor increase. Cases with no information were not reported.

^c^ Cases with complex mutations are listed by each individual FGFR mutation present in the patient.

## RESULTS

### Cohort Description

The somatic evolution of cancer cells leads to an enrichment of functional mutations in sequencing cohorts. For FGFRs, this is exemplified by the frequent detection of hotspot mutations with activating function in sequencing data sets, but despite their relative abundance, FGFR hotspot mutations only make up a small fraction of the FGFR mutational landscape observed in tumors (Data Supplement, Fig S1). We used the large data sets of COSMIC to identify an analysis set of 65 recurrent mutations located outside of the kinase domains of FGFR1, FGFR2, FGFR3, or FGFR4 and for which no convincing clinical or preclinical data were available at the time of collection (Figs 1B and 1C). We complemented the set with six manually chosen variants based on position and presumed functional properties and 25 control mutations featuring known activating and inactivating mutations.

### High-Throughput Screening of FGFR VUS

**FIG 2.**
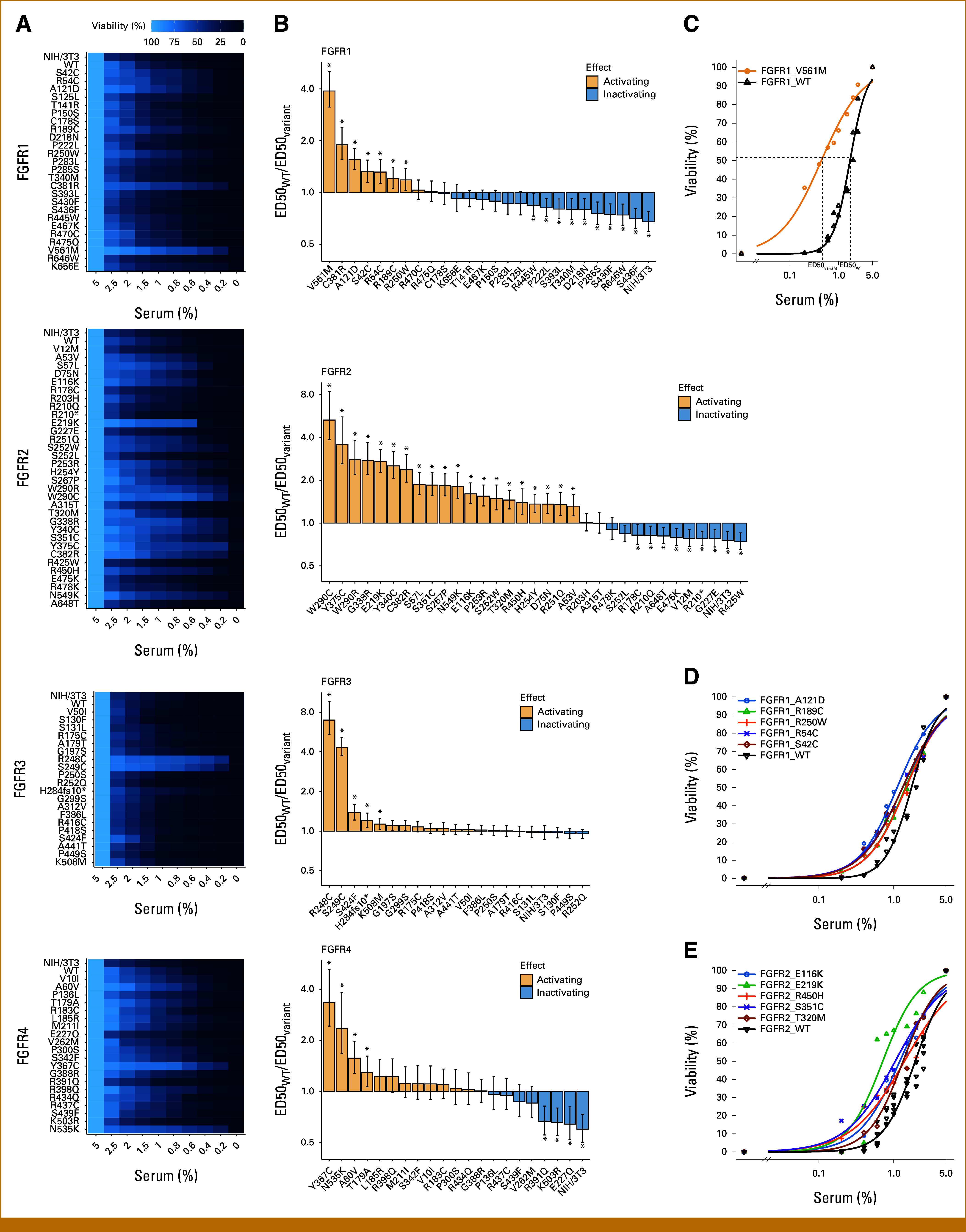
(A) High-throughput viability screening of NIH/3T3 expressing FGFR variants. (B) Ratio of ED50 for serum of NIH/3T3 expressing FGFR variants compared with FGFR wild-type proteins. (C) Determination of ED50 for serum as a measure of growth factor dependency. The example shows NIH/3T3 FGFR1 V561M and NIH/3T3 FGFR1 WT. (D and E) Viability of NIH/3T3 expressing selected activating nonkinase domain variants of FGFR1 (D) or FGFR2 (E). Error bars indicate 95% CI. Asterisks mark variants with significantly different ED50 (*P* < .05, Benjamini-Hochberg adjusted) compared with the corresponding FGFR wild-type. FGFR, fibroblast growth factor receptor; WT, wild type.

NIH/3T3 fibroblasts are a well-studied model system and can be readily transformed with mutationally activated FGFRs^21,56^ leading to increased proliferation, transformed cell shape, reduced growth factor dependency, anoikis resistance, and increased motility.^21,56^ We cloned and overexpressed FGFR variants in NIH/3T3 and conducted a high-throughput screening experiment (HTS) to test for reduced serum dependency of the transformed cell lines (Fig 2). Sixty-nine cell lines including most of the control variants showed a significantly altered dependency on growth factors compared with cells overexpressing the respective FGFR wild-type protein. We quantified the magnitude of effects by calculating the ratio of ED50s for serum between cells carrying FGFR mutations and their wild-type counterparts (Fig 2B). Known activating mutations demonstrated strongly reduced growth factor dependency validating the screening approach. We noticed that the reduction in growth factor dependency provided by overexpression of FGFR wild-type proteins differs considerably. In particular, FGFR4 wild-type overexpression exerts pronounced effects, whereas FGFR3 wild-type overexpression had little impact.

### Validation of Hits From HTS and Functional Assays

The screening conditions in our HTS required a compromise to capture variants with different impact, and we observed that some known activating variants performed weaker than expected. We thus validated all hits from the primary screen in an independent laboratory setting adapting the screening conditions for some strongly activating variants to better capture their impact (Data Supplement, Fig S2). The validation experiments confirmed the activating characteristics of the majority of VUS hits from the primary screen and also clearly recapitulated known characteristics of strongly activating variants such as FGFR1 K656E. Hits from the HTS that were not confirmed in the validation experiments were considered to be neutral or inactivating.

**FIG 3.**
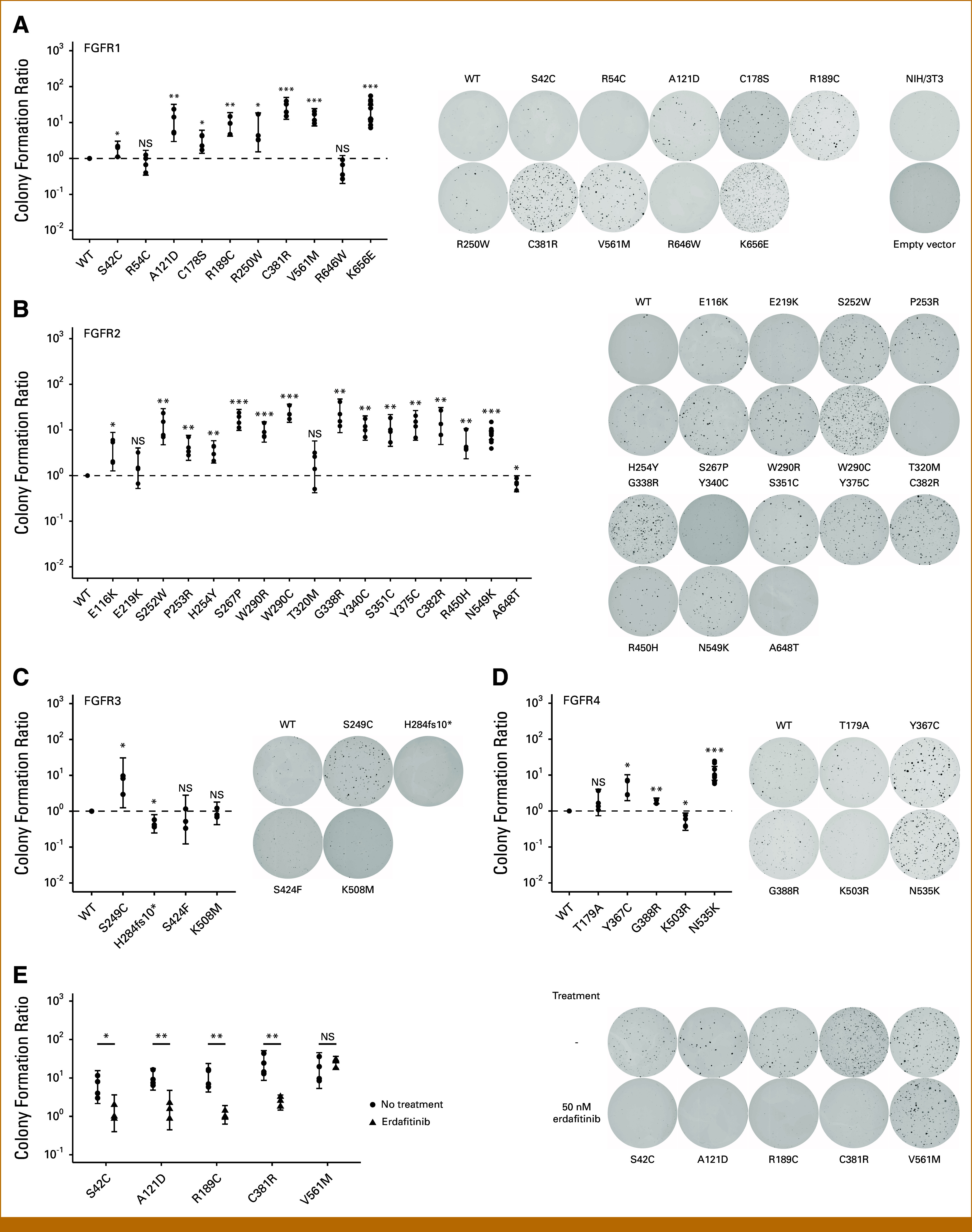
(A-D) Colony formation ratio and representative images of soft agar assays of NIH/3T3 expressing variants of FGFR1 (A), FGFR2 (B), FGFR3 (C), or FGFR4 (D). Statistical indicators show comparison with a ratio of 1 (wild-type, dashed line). (E) Soft agar assays and representative images of NIH/3T3 expressing variants of FGFR1 with or without 50 nM erdafitinib. Statistics: two-tailed *t*-test. Error bars indicate 95% CI. NS, not significant; WT, wild type. *P < .05, **P < .01, ***P < .001.

FGFR alterations can imbue anoikis resistance, which is considered a strong indicator of malignancy and can be assessed in clonogenic or soft agar assays that test the potential of cells to grow into isolated colonies without support by a solid surface.^57,58^ We conducted soft agar assays of NIH/3T3 expressing activating FGFR variants (Fig 3). Increased colony formation underscored the potential of FGFR variants to act as drivers (Figs 3A-3D). Addition of erdafitinib reversed the effects demonstrating that FGFR variants are the causative agent for the observed cellular transformation, whereas the known resistance variant FGFR1 V561M maintained its transforming effect in the presence of erdafitinib (Fig 3E).

### Comparison With Predictions by AI Models and Available Clinical Benefit Data

Computational tools are increasingly used to support the interpretation of somatic variants. The number of available tools and their predictive power have increased rapidly in the past years. To compare our experimental results with computational variant prediction, we selected three state-of-the-art AI models: AlphaMissense, trained for pathogenicity prediction of germline variants incorporating structural context,^27^ PrimateAI-3D, trained on tolerated germline mutations across different primate species,^28^ and CancerVar Oncogenic Prioritization by Artificial Intelligence (OPAI), a prediction tool specifically built for variant prediction in cancer.^59^ Of note, although AlphaMissense and PrimateAI-3D were trained for pathogenicity prediction of germline variants, their predictions may still be valuable for judging potentially damaging effects of somatic variants because cancer driver mutations are mostly depleted from the germline.^60^

**FIG 4.**
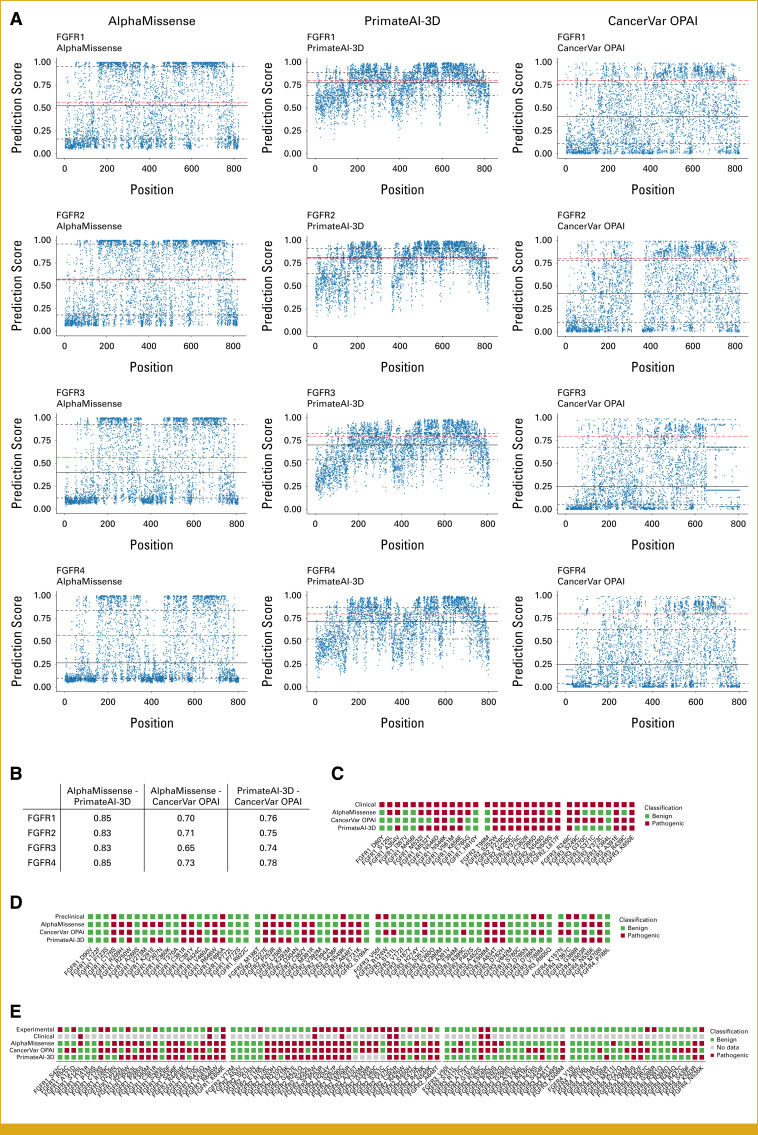
(A) Prediction scores produced by AlphaMissense, PrimateAI-3D, and CancerVar OPAI for FGFR1, FGFR2, FGFR3, and FGFR4. Red dashed lines indicate the threshold used for classification of variants, solid black lines the median of scores, and dashed black lines the upper and lower quartile. (B) Spearman correlations of AI model predictions. (C-E) Comparison of AI predictions with clinical benefit data (C), the preclinical test set (D), and the FGFR variants is experimentally assessed in this study (E). AI, artificial intelligence; FGFR, fibroblast growth factor receptor; OPAI, Oncogenic Prioritization by Artificial Intelligence.

For improved comparability, we limited the analysis to unique nonsynonymous, non-nonsense protein variants resulting from single-point mutations. We first explored the prediction landscape for all FGFR variants matching the criteria and noted profound differences in the distribution of scores between the models (Fig 4). Despite the differences, Spearman correlations indicated a moderate to strong concordance for prediction scores produced by the models (Fig 4B; Data Supplement, Figs S3A and S3B). For classification, we used precalibrated classifications provided by AlphaMissense,^27^ a threshold of 0.8 for PrimateAI-3D following the authors' recommendations, and adopted this threshold for CancerVar OPAI based on the high correlation of prediction scores between the models. Mutations below the respective pathogenicity thresholds were classified as benign enforcing binary decisions.

Evaluating the usefulness of predictions for clinical use requires a clinical comparison set. We conducted a comprehensive literature research on clinical cases of patients who exhibited tumors with FGFR mutations and experienced clinical benefit upon FGFR-targeted therapy (Table 1). We compared predictions of the models with the clinical benefit data and found only modest performance of the AI models (Fig 4C). The sensitivity of all three models was 63% (95% CI, 0.45 to 0.77). We noticed that AlphaMissense and PrimateAI-3D had difficulties with recapitulating the pathogenicity of some well-known extracellular target mutations in FGFR3, for example, FGFR3 S249C, FGFR3 G370C, and FGFR3 Y373C. CancerVar OPAI, on the other hand, demonstrated difficulties with classifying FGFR1 mutations. Notably, restricting the set of FGFR mutations to mutations with at least one exceptional responder (progression free survival >9 months) in a clinical setting leads to markedly improved sensitivity of AI model predictions (Data Supplement, Fig S4), which possibly indicates that the models are capable of recapitulating most strong and well-known effects but fail to accurately predict effects of other mutations.

We then explored the concordance of the AI model predictions with preclinical data. We compiled a test set featuring FGFR variants from the FASMIC database^30^ which provides annotations based on two cell line models other than NIH/3T3. The test set was complemented with known function-impairing and gain-of-function mutations. Mutations already present in the clinical set were excluded. The performance of the models was comparable with the clinical benefit test set with sensitivity ranging from 50% to 67% and specificity between 57% and 65% (Fig 4D). ROC curves drawn for the preclinical data set indicate that the selected thresholds for classification were reasonably chosen to maintain a balance between sensitivity and specificity (Data Supplement, Figs S3C and S3D).

Finally, we compared the results from our in vitro experiments with the predictions of the AI models and the clinical benefit data (Fig 4E). Eight of nine mutations with known clinical benefit to FGFR-targeted therapy were correctly identified as activating mutations in our experiments, the exception being FGFR1 S125L that showed no activating effect in the HTS. Using the experimental data as ground truth, the AI models reached only sensitivities between 67% and 78% and specificities between 45% and 62%.

### Sensitivity of FGFR Variants to Tyrosine Kinase Inhibitors

**FIG 5.**
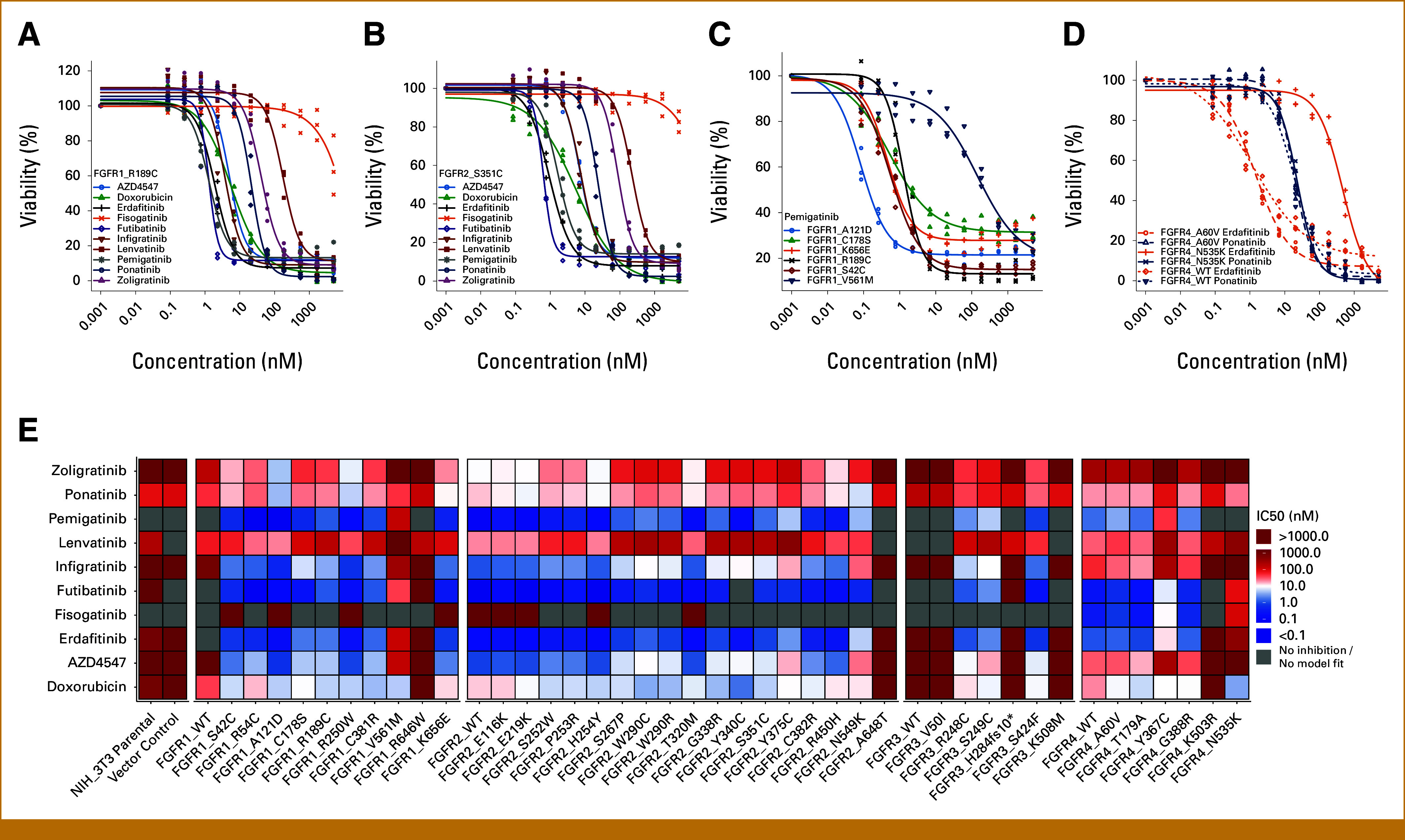
(A and B) Dose-response curves of NIH/3T3 expressing FGFR1 R189C or FGFR2 S351C treated with doxorubicin or FGFR-targeting drugs. (C) Dose-response curves of NIH/3T3 expressing selected FGFR1 variants treated with pemigatinib. (D) Dose-response curves of NIH/3T3 expressing selected FGFR4 variants treated with erdafitinib or ponatinib. (E) Heat map showing IC50 determined from dose-response models of NIH/3T3 expressing FGFR variants treated with doxorubicin or FGFR targeting drugs. FGFR, fibroblast growth factor receptor.

To establish the sensitivity profiles of variants, we conducted high-throughput drug screens testing nine FGFR targeting inhibitors on cell lines expressing FGFR VUS or control variants (Fig 5). Screening conditions were chosen to require the presence of an activating mutation supporting growth of cells. Doxorubicin was included as a nontargeted agent with cytotoxicity to proliferating cells. Cells expressing FGFR variants with inactivating effect and NIH/3T3 parental cells showed little to no growth reflected by low observed inhibition by both doxorubicin and targeted agents. Cells expressing activating FGFR variants, on the other hand, were strongly inhibited by doxorubicin and showed differential inhibition by targeted drugs. The approved pan-FGFR inhibitors futibatinib, erdafitinib, and pemigatinib efficiently inhibited FGFR variants across FGFRs (Fig 5E). In general, nonkinase domain variants such as FGFR1 R189C (Fig 5A) or FGFR2 S351C (Fig 5B) were strongly inhibited by all FGFR TKIs. TKIs with a broad target spectrum like ponatinib and lenvatinib also inhibited cells expressing activating FGFR variants but typically at much higher IC50 than specific FGFR inhibitors. We included FGFR1 V561M as an exemplary resistance mutation (Fig 5C). None of the investigated FGFR VUS displays a comparable resistance effect like FGFR1 V561M supporting the view that nonkinase domain FGFR mutations are not associated with resistance to FGFR TKIs. We included some variants in the drug screen for which validation experiments had not confirmed activating potential (FGFR2 E219K, FGFR2 T320M, FGFR3 V50I, FGFR3 H284fs10*, FGFR4 A60V) and found that their profiles resembled their FGFR wild-type counterparts (Figs 5D and 5E). We noted that the FGFR4-specific inhibitor fisogatinib demonstrated excellent selectivity with little inhibition for cells not expressing functional FGFR4. However, fisogatinib failed to efficiently inhibit the FGFR4 hotspot variant FGFR4 N535K (Fig 5D). The only inhibitor included in this screen capable of efficiently targeting FGFR4 N535K was ponatinib. Both the generally low sensitivity of FGFR4 N535K to TKI inhibition^21^ and the possibility to inhibit FGFR4 N535K with ponatinib have been described independently,^61^ and their direct comparison in our experiments confirms ponatinib as a potentially superior substance for treatment of FGFR4 N535K.

## DISCUSSION

The rapid discovery of novel variants through next-generation sequencing has outpaced our ability to accurately assign interpretations to these variants. The variant interpretation problem plagues molecular pathologists and oncologists in the assessment of both germline variants and somatic alterations in cancer. Experimental approaches such as functional assays or computational assessments using in silico predictions have been proposed to address this issue,^62,63^ and we designed our study to allow comparison of different approaches.

We chose to characterize FGFR VUS in NIH/3T3, a cell line model that has been shown to have strong concordance of in vitro experimental results with observations from animal studies and clinical cases with FGFR alterations.^21,42,51,64^ In our experiments, NIH/3T3 correctly recapitulated the clinical benefit experienced by patients treated with FGFR inhibitors for eight of nine mutations showing both their activating potential and druggability. However, NIH/3T3 did not show activating potential of FGFR1 S125L, potentially in contrast to the clinical benefit observed in one case.^34^ To verify this finding, we reassayed NIH/3T3 FGFR1 S125L in an independent laboratory setting and observed minimally reduced serum dependency but no significantly increased colony formation, making it a borderline case (Data Supplement, Fig S5). The collected data for this variant agree with results reported in a previous study using NIH/3T3 and thus possibly reflect a limitation of the model system.^21^ On the other hand, there is limited experimental evidence demonstrating meaningful activating potential of FGFR1 S125L with only one study showing sensitivity of cell lines bearing FGFR1 S125L to MEK inhibition.^65^

A general limitation of our experimental model system stems from the use of overexpression constructs leading to elevated protein levels and increased signaling capacity. This effect results in the decreased serum dependency of NIH/3T3 expressing FGFR wild-type proteins compared with untransformed cells. To compensate for this baseline effect, we have compared NIH/3T3 expressing FGFR variants with cells expressing the respective wild-type protein throughout this study. Another fundamental limitation of NIH/3T3 is its incapability to reflect tissue-specific effects of oncogenic mutations. Investigating such interactions requires more complex and less scalable model systems such as patient-derived organoid models or cell lines.

Computational approaches for variant prediction have the inherent advantage of rapid result availability but are to date considered less reliable than experimental approaches as the complexities of biology can lead to unexpected or novel interactions not reflected in computational predictions.^66^ We compared clinical outcomes and experimental data with pathogenicity predictions by state-of-the-art AI models and conclude that such methods are not yet sufficiently developed to reliably predict pathogenicity of variants or even possible clinical responses. In fact, usage of AI models alone could be grossly misleading as demonstrated by the inability of AlphaMissense and PrimateAI-3D to recapitulate approved target mutations in FGFR3. Our analysis is slightly confounded by mutations originating from complex cases with more than one FGFR mutation in a tumor (Table 1), but this fraction is very small and the appearance of complex mutations in the analysis reflects the reality of decision making in a molecular tumor board. Another obstacle for the use of AI predictions for variant interpretation arises from the inability of the investigated models to discriminate between activating and loss-of-function mutations as the models were trained to predict the general pathogenicity of mutations. Although both types of mutations are rightfully considered pathogenic, TKIs can only be effective when targeting activating mutations. The development of function-inferring models would thus be highly beneficial for somatic variant interpretation.

The experiments conducted by us do not elucidate potential mechanisms of FGFR activation, but the collected data warrant discussing existing hypothesis. The extracellular domains of FGFR1, FGFR2, and FGFR3 are known to contribute to autoinhibition and have a destabilizing effect on receptor dimerization in the absence of ligand.^67^ Mutations in the extracellular domains could interfere with inhibitory mechanisms or weaken repulsive interactions. We would expect that the mutational spectrum of such a mechanism would resemble other loss-of-function signatures: a landscape of scattered low-frequency point mutations and few enriched hotspot mutations with more fundamental biological effects. We suggest that such a loss-of-function mechanism could at least partially explain the clinically observed mutational landscape and the high prevalence of moderately activating FGFR1 and FGFR2 mutations detected in our screening experiments (Fig 2).

FGFR small variants are widespread in cancer, but clinical benefits of FGFR targeting have been demonstrated only for a limited set of hotspot mutations with strong activating effects. We show here that many nonkinase domain mutations recurrently found in cancers exert activating effects in vitro and are targetable by FGFR TKIs. The majority of activating variants characterized in this study does not reach levels of activation comparable with well-characterized hotspot mutations, warranting some caution to avoid overinterpretation of results. We note, however, that many of the modestly activating mutations in FGFR2 have similarly strong in vitro effects like FGFR2 S252W, a well-characterized mutation with several published cases of patients experiencing clinical benefit from FGFR TKIs. We therefore propose to consider the newly discovered activating variants as candidate FGFR mutations for clinical evaluation in the context of clinical trials and beyond.
